# Intrathoracic Goiter Treated Through a Cervical Approach: A Case Report

**DOI:** 10.7759/cureus.87603

**Published:** 2025-07-09

**Authors:** Nora Lis Flores-Olmos, Rocio Prieto-Ramos, Daniel Alejandro Gamón Briseño, Martha Alicia Cornelio Hernández, Roberto Armando Gutiérrez Ceballos, Daniel Eduardo Luna López

**Affiliations:** 1 Department of General Surgery, Hospital Regional Dr. Valentin Gomez Farias ISSSTE (Instituto de Seguridad y Servicios Sociales de los Trabajadores del Estado), Zapopan, MEX; 2 Department of General Surgery, Hospital General ISSSTE (Instituto de Seguridad y Servicios Sociales de los Trabajadores del Estado), Zacatecas, MEX; 3 Department of General Surgery, Hospital Regional Dr. Valentín Gómez Farías ISSSTE (Instituto de Seguridad y Servicios Sociales de los Trabajadores del Estado), Zapopan, MEX; 4 Department of Surgery, Hospital General de Occidente, Zapopan, MEX

**Keywords:** cervical surgery, ectopic thyroid, intrathoracic goiter, mediastinal mass, thyroidectomy, thyroid tissue

## Abstract

Intrathoracic goiter (ITG), also referred to as retrosternal or mediastinal goiter, is an uncommon clinical entity characterized by the presence of thyroid tissue within the mediastinum, either as an extension of a cervical goiter or as primary ectopic tissue. Its diagnosis may be challenging due to its variable presentation and potential to mimic other thoracic pathologies. We present the case of a 41-year-old female with progressive dyspnea and an anterosuperior mediastinal mass initially suspected to be a thymoma. A percutaneous biopsy revealed thyroid tissue, and complete resection was achieved through a cervical approach. Histopathological examination confirmed a goiter with follicular hyperplasia and no evidence of malignancy. This case highlights the importance of including ITG in the differential diagnosis of mediastinal masses and supports the efficacy of the cervical approach as a safe surgical option in selected patients, avoiding more extensive thoracic procedures.

## Introduction

Intrathoracic goiter (ITG), also referred to as retrosternal, mediastinal, or subclavicular goiter, is a rare and complex clinical entity characterized by the partial or complete presence of thyroid tissue within the mediastinum [1]. Since its first anatomical description by Haller in 1749, multiple definitions have been proposed, contributing to heterogeneity in its recognition and to variability in its reported prevalence, which ranges from 0.2% to 6% of all goiters depending on the diagnostic criteria used [1-3]. Despite its low frequency, accurate identification is essential due to the potential clinical and surgical implications.

Over time, ITG has been the subject of various terminological interpretations and clinical classifications, resulting in considerable inconsistency in its conceptualization within the medical literature [3,4]. In particular, the distinction between primary and secondary forms remains a topic of ongoing analysis and discussion from both embryological and surgical perspectives [1,4,5].

In recent decades, the development of more standardized surgical techniques and the widespread availability of advanced imaging studies have led to an increase in its detection, either as an incidental finding or in the context of mediastinal evaluations for other causes [2,6]. Although infrequent, ITG remains clinically relevant not only due to its potential to cause complications but also because of its capacity to mimic other thoracic diseases, such as thymomas or lymphomas, which underscores the importance of timely recognition [1,2].

## Case presentation

**Figure 1:**
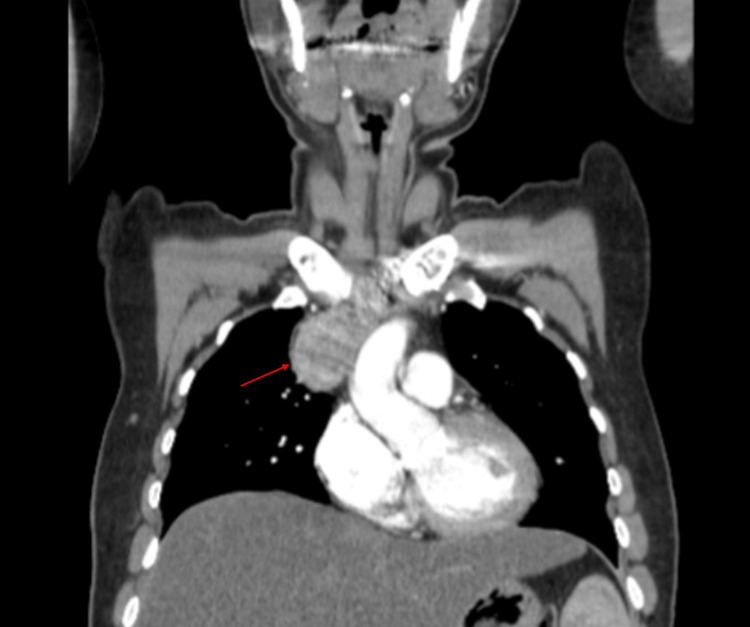
Contrast-enhanced computed tomography, coronal view, showing a mass in the superior mediastinum measuring approximately 5 x 8 cm, with no enhancement after contrast administration.

A 41-year-old female patient with a history of type 2 diabetes mellitus under medical treatment presented in September 2024 with progressive dyspnea. Imaging studies incidentally revealed an anterosuperior mediastinal mass located above the aortic arch, initially suspected to be a thymoma (Figure 1). A percutaneous biopsy reported thyroid tissue with a normofollicular pattern, and surgical resection with histopathological and immunohistochemical analysis was recommended. The case was referred to general surgery and cardiothoracic surgery for a combined surgical approach, with ectopic mediastinal thyroid tissue considered in the differential diagnosis.

**Figure 2:**
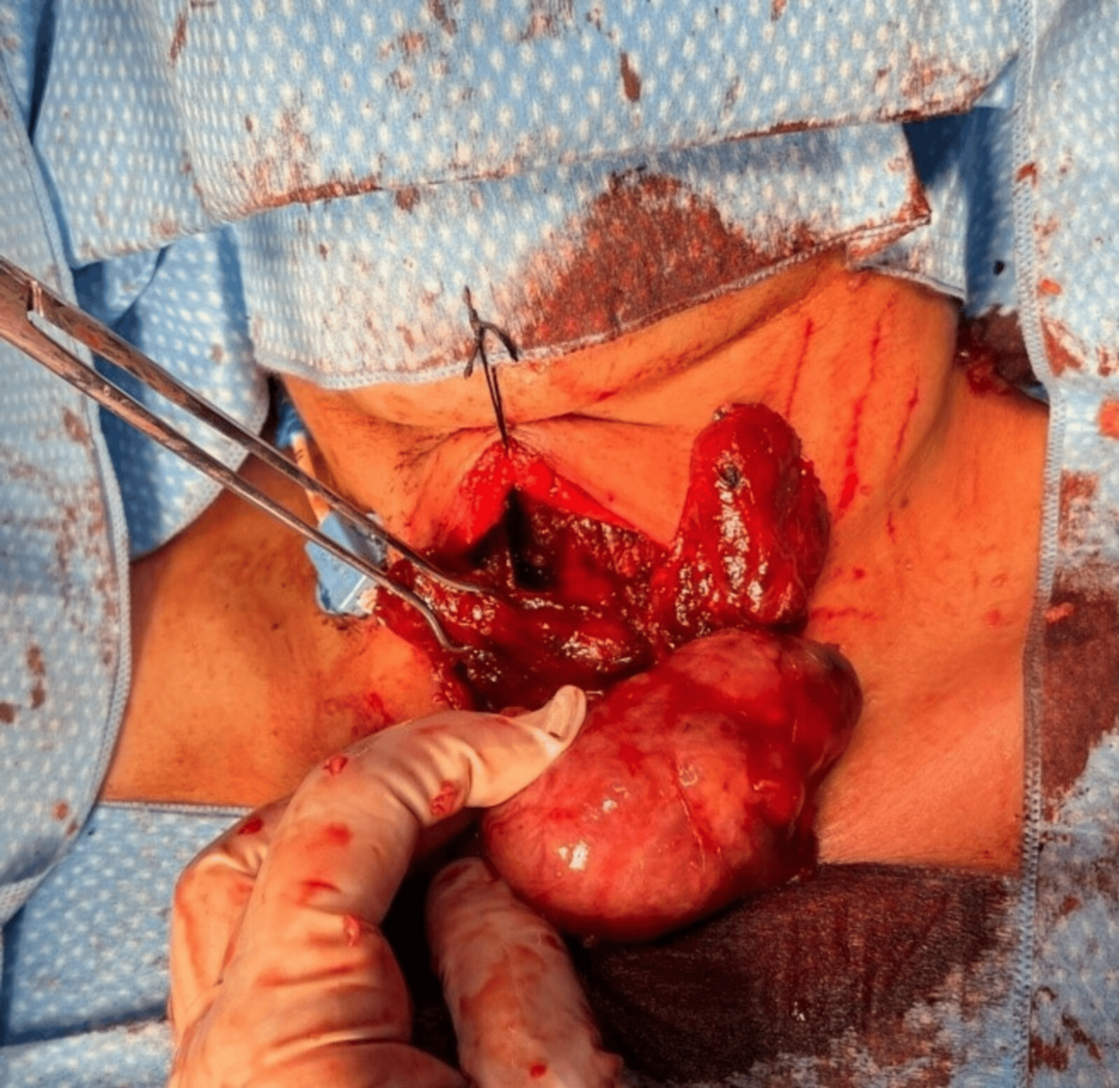
Surgical exposure of both thyroid lobes and the mediastinal mass through a cervical incision

**Figure 3:**
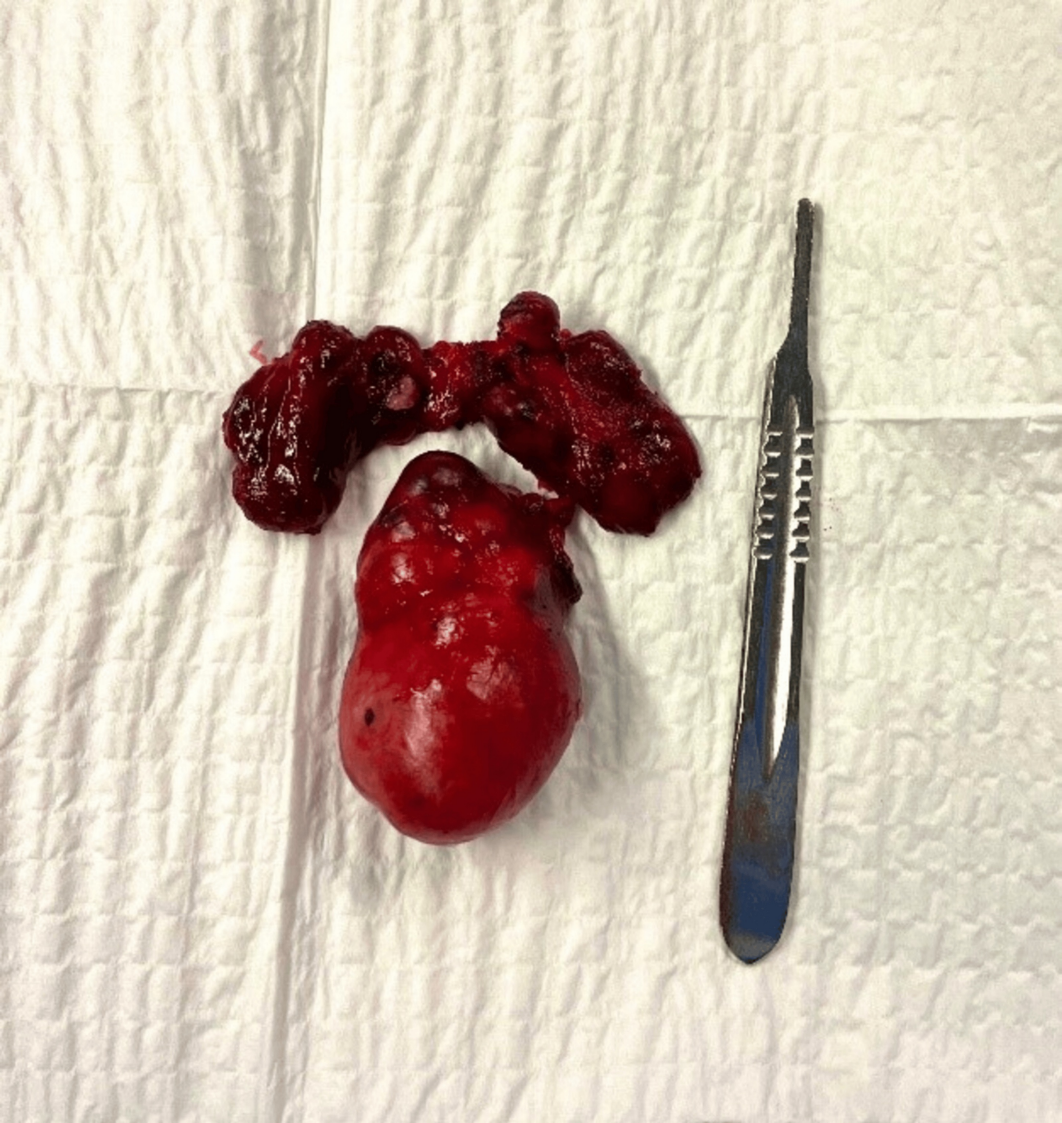
Pathological specimen removed during the surgical procedure

During the surgical procedure, the thyroid gland appeared grossly normal, with no significant macroscopic abnormalities. However, a retrosternal mass measuring 5 x 8 cm was identified in the superior mediastinum and was completely excised through a cervical approach, avoiding the need for sternotomy or thoracotomy (Figure 2). Histopathological examination of the resected tissue revealed thyroid tissue with follicular hyperplasia, consistent with goiter. The thyroidectomy specimen also showed a 0.4 x 0.3 cm follicular adenoma in the lower half of the right thyroid lobe, along with adenomatoid changes and follicular hyperplasia in the remaining parenchyma. No malignancy was identified (Figure 3).

## Discussion

Embryologically, the thyroid gland develops from the fusion of the medial and lateral thyroid anlages. During its descent from the base of the tongue to its final position anterior to the trachea, remnants of ectopic thyroid tissue may persist along the migratory path. In rare cases, these remnants are located within the thoracic cavity, giving rise to what is referred to as primary intrathoracic goiter [1]. In contrast to the secondary type, which results from the downward extension of a cervical goiter, the primary form lacks anatomical continuity with the cervical thyroid gland and derives its blood supply from intrathoracic vessels [1,2,4].

Intrathoracic goiter is frequently associated with multinodular goiter. Although often asymptomatic, it may present clinically with compressive symptoms, such as dyspnea, dysphagia, stridor, or superior vena cava syndrome, due to its proximity to mediastinal structures [2,3]. Its prevalence is higher in women during the fifth and sixth decades of life and may be influenced by geographic, genetic, and environmental factors, including iodine intake [2].

Computed tomography (CT) is the most useful diagnostic tool, as it allows for the assessment of the goiter’s extent and its relationship with adjacent structures and facilitates surgical planning [3,4]. While management options may include observation, radioactive iodine therapy, or surgery, in symptomatic cases or when the etiology is uncertain, surgical resection remains the treatment of choice [1,3,4].

The cervical approach is considered the standard initial technique in most cases, particularly for secondary goiters with continuity to the cervical thyroid [5]. This approach provides adequate exposure for tissue dissection and favors the preservation of key structures such as the recurrent laryngeal nerves and parathyroid glands. It is also associated with lower morbidity and faster recovery compared to thoracic approaches.

However, the success of the cervical approach depends on the size, location, and degree of adherence of the goiter. In complex cases, sternotomy or thoracotomy may be required to achieve complete and safe resection [5,6]. Detailed preoperative imaging is essential to anticipate surgical challenges and avoid complications, particularly in primary goiters with intrathoracic vascular supply [5,6].

This case highlights the importance of recognizing this rare entity, its diagnostic and therapeutic implications, and demonstrates that the cervical approach can be a safe and effective option in selected patients.

## Conclusions

Intrathoracic goiter is an uncommon but clinically significant condition. Timely diagnosis is essential due to the risk of compressing vital mediastinal structures. This case underscores the importance of including intrathoracic goiter in the differential diagnosis of mediastinal masses, particularly when imaging and histopathological findings are suggestive of thyroid tissue. The cervical approach enabled complete and safe resection without the need for major thoracic procedures, reinforcing its value as a viable surgical option in appropriately selected patients. Ultimately, successful management depends on a multidisciplinary approach and a thorough understanding of the anatomical and embryological variations of the thyroid gland.
